# Editorial

**Published:** 1871-03

**Authors:** 


### Editorial.

Medical Society of District of Columbia.—This Society, at its annual meeting, elected Dr. J. M. Toner President.

Dr. Toner is one of the most meritorious men in the profession. Industrious, scholarly, dignified, and blessed with a large amount of practical good sense, he will do honor to the position in which he is placed. His inaugural address was filled with interesting statistics in relation to the past history of the Society and the present condition of the profession in the District.

Good Appointment.—We see that our friend and former pupil, H. C. Snitcher, M.D., of Wilmington, Delaware, has been appointed to a chair in one of the educational institutions of that city, and is now lecturing on Physiology and Hygiene.

---

Chloroform Deaths.—In the January number of the Cincinnati *Lancet and Observer* is a valuable article on deaths from chloroform, by Dr. Dawson, of that city. The writer quotes a table of 208,893 cases of anæsthesia, by different anæsthetics, with the ratio of deaths from each; and says he found it in the "Eclectic Department" of the Louisville and Richmond *Medical Journal*, without the name of the author or the source from which it originally came. We would inform Dr. Dawson, and all others, that the article from which he quotes, in the Louisville and Richmond *Medical Journal,* was taken from the Chicago Medical Examiner, and was written by Edmund Andrews, M.D., Professor of Surgery in the Chicago Medical College. The omission of the Louisville *Journal* to give proper credit was, doubtless, an oversight, but it was rather singular that the name of the author should also be omitted.

College Commencement Exercises.—The public exercises accompanying the conferring of degrees and the Annual Meeting of the Alumni, at Rush Medical College, took place on the first day of February, and was an occasion of interest and enjoyment to the friends of the school. We notice that the number of graduates was considerably less than for several years past.

We noticed in the proceedings another item which may have significance in connection with the fact just stated.

One of the members of the Faculty, during some part of the proceedings, distinctly advocated placing the College on such a basis that the "Lecture fees" could be abolished, and medical instruction made free. Let those who think the *dignity* of the profession depends upon the amount of college fees exacted of the students, note the signs of the *times*.

---

Chicago Woman's Hospital Medical College.—This new institution held its First Annual Commencement in the afternoon of the 23d of February, when the degree of doctor of medicine was conferred on three females who had complied with all the conditions usually required of medical students.

---

Chicago Medical College—Medical Department N. W. University.—The Annual Commencement Exercises of this College will take place on Monday and Tuesday, March 13th and 14th, 1871, in the hall of the College, on the corner of Prairie Avenue and Twenty-sixth Street. The exercises on Monday will commence at 2 o'clock P.M. and continue until 5 o'clock, and will consist of a public examination of the candidates for graduation, and reading of theses. Members of the profession generally are especially invited to attend on that day. The exercises on Tuesday will commence at 2½ o'clock P.M., and will include the usual programme. It is expected that Dr. Haven, President of the Northwestern University, will preside and confer the degrees. The regular valedictory address will be delivered by Prof. R. N. Isham. The public, as well as the profession, are cordially invited to attend.

Meeting of Alumni Association of Chicago Medical College.—We are informed that the Annual Meeting of this Association will be held in the College, at 10 o'clock A.M. of Monday, March 13, 1871, instead of Tuesday as stated in our last issue. Annual address by the President, Dr. J. M. Woodworth.

---

Medical Association of East Tennessee.—The Medical Association of East Tennessee was revived at a meeting held at Knoxville February 2, 1871.

The following were elected officers for the ensuing year:

*President*—Dr. F. K. Bailey.

*Vice-Presidents*—Drs. James Rodgers and J. M. Boyd.

*Recording Secretary*—Dr. M. A. Alexander.

*Corresponding Secretary*—Dr. S. D. Moses.

*Treasurer*—Dr. S. M. Burnett.

The President, Dr. Bailey, is one of our old and most valued friends.

We are glad to see him working for the organization of the profession, and honored in his work.

---

### AMERICAN MEDICAL ASSOCIATION.

---

Office of Perm't Sec'y, Wm. B. Atkinson, M.D.,

*1400 Pine Street, Philadelphia.*

The Twenty-second Annual Session will be held in San Francisco, Cal., May 2, 1871, at 11 A.M.

The following committees are expected to report:

*On Cultivation of the Cinchona Tree*—Dr. Lemuel J. Deal, Pennsylvania, Chairman.

*On Inebriate Asylums*—Dr. C. H. Nichols, D.C., Chairman.

*On Institutions for Inebriates*—Dr. Joseph Parrish, Pennsylvania, Chairman.

*On the Structure of the White Blood Corpuscles*—Dr. J. G. Richardson, Pa., Chairman.

*On Vaccination*—Dr. Henry A. Martin, Mass., Chairman.

*On the Comparative Merits of Syme's and Pirogoff's Operations*—Dr. Geo. A. Otis, U.S.A., Chairman.

*On Lithotrity*—Dr. E. M. Moore, New York, Chairman.

*On Veterinary Medicine*—Dr. Samuel D. Gross, Pa., Chairman.

*On Protest of Naval Surgeons, Etc.*—Dr. W. S. W. Ruschenberger, U.S.N., Chairman.

*On National Medical School*—Dr. Francis Gurney Smith, Pa., Chairman.

*On American Medical Association Journal*—Dr. J. P. White, New York, Chairman.

*On Criminal Abortion*—Dr. D. A. O'Donnell, Maryland, Chairman.

*On Nomenclature of Diseases*—Dr. Francis Gurney Smith, Pa., Chairman.

*On National System of Quarantine*—Dr. J. C. Tucker, Cal., Chairman.

*On What, if any, Legislative Means are Expedient and Advisable to Prevent the Spread of Contagious Diseases*—Dr. M. H. Henry, New York, Chairman.

*On Renewal of Prescriptions by Apothecaries without Authority*—Dr. R. J. O'Sullivan, New York, Chairman.

*On American Medical Necrology*—Dr. C. C. Cox, D.C., Chairman.

*On Medical Education*—Dr. Ely Geddings, South Carolina, Chairman.

*On Medical Literature*—Dr. P. G. Robinson, Mo., Chairman.

*On Prize Essays*—Dr. T. M. Logan, California, Chairman.

*On the Climatology and Epidemics of*—Maine, Dr. J. C. Weston; New Hampshire, Dr. P. A. Stackpole; Massachusetts, Dr. H. I. Bowditch; Rhode Island, Dr. C. W. Parsons; Connecticut, Dr. J. C. Jackson; New York, Dr. W. F. Thoms; New Jersey, Dr. C. F. J. Lehlbach; Pennsylvania, Dr. D. F. Condie; Maryland, Dr. C. H. Ohr; Georgia, Dr. Juriah Harriss; Missouri, Dr. F. E. Baumgarten; Alabama, Dr. R. F. Michel; Texas, Dr. S. M. Welsh; Illinois, Dr. R. C. Hamil; Indiana,

Dr. J. F. Hibberd; District of Columbia, Dr. T. Antisell; Iowa, Dr. J. C. Hughes; Michigan, Dr. G. P. Andrews; Ohio, Dr. T. L. Neal; California, Dr. F. W. Hatch; Tennessee, Dr. B. W. Avent; West Virginia, Dr. E. A. Hildreth; Minnesota, Dr. Chas. N. Hewitt; Virginia, Dr. W. O. Owen; Delaware, Dr. L. B. Bush; Arkansas, Dr. G. W. Lawrence; Mississippi, Dr. J. P. Moore; Louisiana, Dr. S. M. Bemiss; Wisconsin, Dr. J. K. Bartlett; Kentucky, Dr. L. P. Yandell, Sr.; Oregon, Dr. E. R. Fisk; North Carolina, Dr. W. H. McKee.

Secretaries of all medical organizations are requested to forward lists of their delegates as soon as elected, to the Permanent Secretary.

Any respectable physician who may desire to attend, but cannot do so as a delegate, may be made a *member by invitation*, upon the recommendation of the Committee of Arrangements.

W. B. ATKINSON.

---

OBITUARY.—Report of the committee of DeWitt County Medical Society, on the death of the late B. K. Shurtleff:

WHEREAS, In the dispensation of an all-wise Providence, it has become our painful duty to notice the death of a worthy and beloved member of this Society, to-wit: Benjamin K. Shurtleff, M.D., who died on the 18th day of December, 1870, and,

WHEREAS, In token of the high estimate the members of this Society, and the community generally, has placed upon the character and usefulness of our deceased fellow member and co-laborer in the science of medicine, and also, because we deemed it due to our own feelings as men and physicians, that this Society should indicate its respect for the deceased in such form as to render the manifestation permanent, be it therefore,

*Resolved*, That, in the death of Dr. Shurtleff, this Society has lost a member who was earnest in the acquisition of medical knowledge, apt in the selection of remedies to combat diseases, a close observer of medical ethics, always courteous in his deportment towards his brother physicians, and one who, had not physical disability prevented, would have become a bright star in the profession of his choice.

*Resolved*, That, these preambles and resolutions be spread upon the records of the Society, and that a copy of the same be transmitted to the widow of the deceased, with the assurance that the members of this Society deeply sympathize with her in her great bereavement.

C. GOODBRAKE, M.D., }
W. W. ADAMS, M.D., } *Committee.*
JOHN WRIGHT, M.D., }

---

SEX OF THE FŒTUS IN UTERI DETERMINED BY THE NUMBER OF CARDIAC PULSATIONS.—The *Edinburgh Medical Journla* contains the following instructive and suggestive tables:

The first case was one of twins; the heart of one fœtus was heard in the right groin, beating 110 in the minute, and on delivery it proved to be a male; the second heart was heard in the left hypochondrium, beating 154, and on delivery it was found to be a female.

TABLE I—MALES.

| | | | | | | | | | |
|---|---|---|---|---|---|---|---|---|---|
| 2. | Fœtal | pulsation, | 138 | per minute. | 15. | Fœtal | pulsation, | 116 | per minute. |
| 3. | " | " | 138 | " | 16. | " | " | 120 | " |
| 4. | " | " | 135 | " | 17. | " | " | 120 | " |
| 5. | " | " | 130 | " | 18. | " | " | 138 | " |
| 6. | " | " | 130 | " | 19. | " | " | 125 | " |
| 7. | " | " | 132 | " | 20. | " | " | 140 | " |
| 8. | " | " | 132 | " | 21. | " | " | 140 | " |
| 9. | " | " | 140 | " | 22. | " | " | 137 | " |
| 10. | " | " | 132 | " | 23. | " | " | 140 | " |
| 11. | " | " | 140 | " | 24. | " | " | 141 | " |
| 12. | " | " | 136 | " | 25. | " | " | 122 | " |
| 13. | " | " | 133 | " | 26. | " | " | 120 | " |
| 14. | " | " | 134 | " | | | | | |

TABLE II—FEMALES.

| | | | | | | | | | |
|---|---|---|---|---|---|---|---|---|---|
| 1. | Fœtal | pulsation, | 150 | per minute. | 9. | Fœtal | pulsation, | 140 | per minute. |
| 2. | " | " | 142 | " | 10. | " | " | 152 | " |
| 3. | " | " | 140 | " | 11. | " | " | 140 | " |
| 4. | " | " | 150 | " | 12. | " | " | 143 | " |
| 5. | " | " | 144 | " | 13. | " | " | 144 | " |
| 6. | " | " | 140 | " | 14. | " | " | 141 | " |
| 7. | " | " | 140 | " | 15. | " | " | 160 | " |
| 8. | " | " | 144 | " | | | | | |

From these two tables it seems that when the pulsation varies from 120 to 140, the probability is that the fœtus will be a male, and when the pulsation varies from 140 to 160, the fœtus will likely be found to be a female. But there are some exceptions to these facts. In three cases in which the pulsation was from 150 to 160, the fœtus proved to be a male; and in fifteen cases in which the pulsation varied from 116 to 138, the fœtuses were found to be females. It therefore appears that there is less frequent variation in the pulsation in the male fœtus than in the female; or rather that there are fewer cases in which the heart's action exceeds 140 in the male, than that it falls below that number in the female.—*Rich. and Louis. Med. Jour.*

### Mortality for the Month of January, 1871.

| | | | | | |
|---|---|---|---|---|---|
| Accidents, drowned | 2 | Diphtheria | 14 | Liver, abscess of | 1 |
| " burned | 3 | Dropsy | 7 | Leucocythæomia | 1 |
| " by fall | 2 | " and peritoneum | 1 | Lungs, congestion | 5 |
| " kicked by horse | 1 | Enteritis | 8 | " hemorrhage | 2 |
| " railroad | 5 | Epilepsy | 2 | Measles | 5 |
| " scalded | 1 | Erysipelas | 4 | " and complications | 2 |
| " suffocation | 1 | Exposure | 2 | Meningitis | 7 |
| Anasarca | 1 | Exhaustion following | | " cerebro spinal | 3 |
| Aneurism of aorta | 1 | child-birth | 1 | " tubercular | 1 |
| Apoplexy | 5 | Fever intermitent | 1 | Metritis, puerperal | 1 |
| Ascites | 2 | " puerperal, | 6 | Occipital bone, depres- | |
| Asphyxia | 1 | " remittent | 2 | sion of | 1 |
| Asthma | 3 | " scarlet | 11 | Old age | 9 |
| Atelectasis pulmonum | 1 | " & complications | 1 | Oedema pulmonum | 1 |
| Bowels, strangulation | | " malignant | 2 | Paralysis | 2 |
| internal | 1 | " typhoid | 8 | Peritonitis | 4 |
| " and stomach neu- | | Gastritis | 2 | Peritonitis puerpural | 2 |
| ralgia of | 1 | Gastro enteritis | 3 | Phlebitis uterine | 1 |
| Brain, congestion of | 2 | Hemorahage, gastric | 1 | Pneumonia | 37 |
| " concussion of | 1 | Hæmoptysis | 1 | " Broncho | 1 |
| " disease of | 1 | Heart disease of | 4 | " and complicatians | 4 |
| " inflammation of | 8 | " atrophy of | 1 | Small-pox | 2 |
| " injury of | 1 | " organic disease of | 2 | Spina bifida | 1 |
| " softening of | 1 | " fatty degeneration | 1 | Spine, caries of | 1 |
| Bronchitis | 6 | " valvular disease | 3 | Stomach, gout of | 1 |
| " capillary | 5 | Hepatitis, | 1 | Suicide by poison | 2 |
| Cancer of breast | 2 | Hernia, umbilical | 1 | Syphilis, congenital | 1 |
| " of side | 1 | Hip-disease | 1 | Tabes mesenterica | 10 |
| " of stomach | 1 | Hydrocephalus | 5 | Teething | 3 |
| " of throat | 1 | " acute | 2 | Throat, ulceration of | 1 |
| Cholera Morbus | 2 | " chronic | 1 | Trichiniasis | 1 |
| Consumption | 51 | Inanition | 14 | Uræmia | 1 |
| Convulsions | 64 | Intussusception | 1 | Uterus, rupture of | 1 |
| Croup | 27 | Kidneys and Liver dis- | | Urinary infilitation | 1 |
| Cyanosis | 2 | ease of | 1 | Vitality deficient | 3 |
| Debility | 3 | Laryngitis | 1 | Whooping-cough | 4 |
| Diabitis | 1 | Leg & arm inflamma- | | | |
| Diarrhœa | 1 | tion of | 1 | Total | 451 |

| | | | | | |
|---|---|---|---|---|---|
| Premature births | 10 | Still births | 56 | Total | 66 |

### AGES.

| | | | | | |
|---|---|---|---|---|---|
| Under 1 | 158 | 10 to 20 | 16 | 70 to 80 | 13 |
| 1 to 2 | 39 | 20 to 30 | 34 | 80 to 90 | 4 |
| 2 to 3 | 20 | 30 to 40 | 49 | 90 to 100 | |
| 3 to 4 | 15 | 40 to 50 | 34 | | |
| 4 to 5 | 13 | 50 to 60 | 15 | Total, | 451 |
| 5 to 10 | 17 | 60 to 70 | 24 | | |

| | | | | | |
|---|---|---|---|---|---|
| Males, | 255 | Females, | 196 | Total, | 451 |
| Single, | 313 | Married | 138 | Total, | 451 |
| White, | 442 | Colored, | 9 | Total, | 451 |

### COMPARISON.

| | | | | | |
|---|---|---|---|---|---|
| Deaths in Jan.,1870, | 451 | Deaths in Jan., 1870, | 483 | Decrease, | 32 |
| Deaths in Dcc., 1870, | 415 | Increase, | 36 | | |

### NATIVITY.

| | | | | | |
|---|---|---|---|---|---|
| Atlantic Ocean | 1 | Denmark | 2 | Norway | 5 |
| Austria | | England | 13 | Russia | 1 |
| Bohemia | 1 | France | 2 | Switzerland | 1 |
| Canada | 2 | Germany | 51 | Scotland, | 8 |
| Chicago, Native | 84 | Holland | | Sweden | 7 |
| Chicago, Foreign | 158 | Ireland | 39 | Wales | 1 |
| U. S., other parts | 73 | Italy | | Unknown | 2 |
| | | | | Total, | 451 |

### MORTALITY BY WARDS FOR THE MONTH.

| Wards. | Mortality. | Wards. | Mortality |
|---|---|---|---|
| 1 | 5 | 18 | 25 |
| 2 | 13 | 19 | 12 |
| 3 | 18 | 20 | 22 |
| 4 | 18 | Accidents | 15 |
| 5 | 11 | County Hospital | 6 |
| 6 | 27 | Erring Woman,s Refuge | 1 |
| 7 | 14 | Home for Friendless | 2 |
| 8 | 36 | Hospital Alexian Bros. | 2 |
| 9 | 42 | Half Orphan Asylum | 1 |
| 10 | 19 | Lake Hospital | 1 |
| 11 | 21 | Marine Hospital | 1 |
| 12 | 18 | Mercy Hospital | 2 |
| 13 | 10 | Scammon Hospital | 1 |
| 14 | 11 | St. Joseph Orphan Asylum | 3 |
| 15 | 28 | Suicide | 2 |
| 16 | 34 | | |
| 17 | 30 | Total, | 451 |

Mean Thermometer, 32⅓°, Rain, 8,77 inches, Deaths daily, 14½.

Receipts from January 25, to February 25.—Dr. H. Nance, Kewanee, Ill., $3.00; Dr. Robert Robson, New Harmony, Ind., 3.00; Dr. John Beale, New Harmony, Ind., 3.00; Dr. D. B. Trimble, City, 3.00; Dr. T. Winston, Foreston, Ill., 4.50; Dr. E. W. Leech, Clifty, Ind., 10.00: Dr. J. T. Frazier, St.Elmo, Ind., 3.00; Dr. J. Schneck, City, 3.00; Dr. R. Woods, City, 6.00; Dr. M. Shepherd, Payson, Ill., 3.00; Dr. G. M. Fox, Lyons, Ill., 10.00; Dr. D. H. Patton, Remington, Ind., 6.00; Dr. W. H. Buchtel, South Bend, Ind., 3.00; Dr. T. D. Fisher, LeRoy, Ind., 6.00; Dr. J. Anderson, City, 3.00; Dr. L. B. Cowles, Caledonia, Minn., 7.00; Dr. T. M. Butler, Pecatonica, Ill., 3.00; Dr. Amos Scott, 10.00; Dr. W. Buchtel, DesMoines, Iowa, 3.00; Dr. E. Andrews, City, 6.00; Dr. C. O'Brien, Edma, Mo., 3.00; Dr. G. Leaming, Ellsworth, Ind., 3.00; Dr. L. D. Tompkins, Cassopolis, Mich., 3.00; Dr. V. L. Hurlbut, City, 3.00; Dr. J. D. Jennings, Sonora, Ill., 3.00; Rev. Geo. Mulfinger, Aurora, Ill. 3.00; Dr. R. Winton, Muncie, Ind., 3.00; Dr. W. T. Knapp, Randall, Ind., 1.50

